# Alert Threshold Algorithms and Malaria Epidemic Detection

**DOI:** 10.3201/eid1007.030722

**Published:** 2004-07

**Authors:** Hailay Desta Teklehaimanot, Joel Schwartz, Awash Teklehaimanot, Marc Lipsitch

**Affiliations:** * Harvard School of Public Health, Boston, Massachusetts, USA;; † Columbia University, New York, New York, USA

**Keywords:** malaria, epidemic detection, early detection, alert threshold, percentile, Ethiopia

## Abstract

We describe a method for comparing the ability of different alert threshold algorithms to detect malaria epidemics and use it with a dataset consisting of weekly malaria cases collected from health facilities in 10 districts of Ethiopia from 1990 to 2000. Four types of alert threshold algorithms are compared: weekly percentile, weekly mean with standard deviation (simple, moving average, and log-transformed case numbers), slide positivity proportion, and slope of weekly cases on log scale. To compare dissimilar alert types on a single scale, a curve was plotted for each type of alert, which showed potentially prevented cases versus number of alerts triggered over 10 years. Simple weekly percentile cutoffs appear to be as good as more complex algorithms for detecting malaria epidemics in Ethiopia. The comparative method developed here may be useful for testing other proposed alert thresholds and for application in other populations.

Accurate, well-validated systems to predict unusual increases in malaria cases are needed to enable timely action by public health officials to control such epidemics and mitigate their impact on human health. Such systems are particularly needed in epidemic-prone regions, such as the East African highlands. In such places, transmission is typically highly seasonal, with considerable variation from year to year, and immunity in the population is often incomplete. Consequently, epidemics, when they occur, often cause high illness and death rates, even in adults (*1**,**2*). The value of timely interventions—such as larviciding, residual house spraying, and mass drug administration—to control malaria epidemics has been documented (*3*), but much less evidence exists about how to identify appropriate times to take such action when resources are limited (*4*). Ideally, public health and vector control workers would have access to a system that provides alerts when substantial numbers of excess cases are expected, and such alerts should be sensitive (so that alerts are reliably generated when excess cases are imminent), specific (so that there are few false alarms or alerts that do not precede significant excess cases), and timely (so that, despite some inevitable delays between sounding the alert and completing interventions, adequate lead time exists to take actions that will reduce cases before they decline "naturally").

A number of such systems have been proposed or implemented, but the comparative utility of these systems for applied public health purposes has not been rigorously established. For example, the World Health Organization has advocated the use of alerts when weekly cases exceed the 75th percentile of cases from the same week in previous years (*5*), and other methods, based on smoothing or parametric assumptions, have also been considered (*6**–**8*). Such methods, known as early detection systems because they detect epidemics once they have begun, can correctly identify periods that are defined by expert observers as epidemic, albeit with varying specificity. However, the ability of early detection systems to generate timely alerts that prospectively identify periods of ongoing excess transmission has not, to our knowledge, been evaluated. A detection algorithm is useful for identifying interventions only if it identifies epidemics at an early phase (*9*), and it (as opposed to prediction) will work only to the extent that epidemics persist (and indeed grow) over time. Thus, detecting unusual cases at one time point will be a reliable indicator that an epidemic is under way (and will be so for long enough that action taken after the warning can still have an effect).

Another approach, known as early warning, attempts to predict epidemics before unusual transmission activity begins, usually by the use of local weather or global climatic variables that are predictors of vector abundance and efficiency, and therefore of transmission potential (*10**–**14*). Such systems have the advantage of providing more advance warning than systems that rely on case counts, but climate- and weather-based systems require data not widely available to local malaria control officials in Africa in real time. Such systems also depend on relatively complex prediction algorithms that may be difficult to implement in the field. Studies of the forecasting ability of such systems are beginning to emerge (*15*); initial studies have focused on the sensitivity rather than on the specificity or timeliness of the alerts.

We describe a method for evaluating the public health value of a system to detect malaria epidemics. We use this method to evaluate several simple early detection systems for their ability to provide timely, sensitive, and specific alerts in a data series of weekly case counts from 10 locations in Ethiopia for approximately 10 years. The fundamental question we address is whether detecting excess cases for 2 weeks in a row, under a variety of working definitions of "excess," can be the basis for a system that anticipates ongoing excess malaria cases in time for action to be taken.

## Materials and Methods

### Study Area and Data

**Figure A1:**
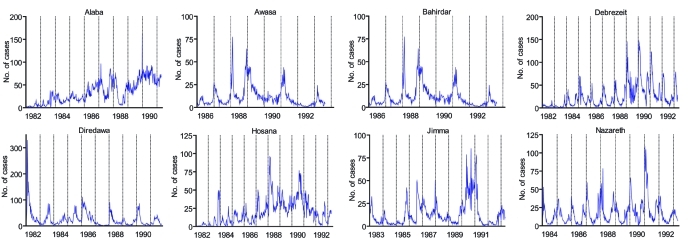
Time series of normalized weekly average daily malaria cases for 10 districts. Years are according to the Ethiopian calendar, in which year y begins on September 11 of year y+7 in the Western calendar.

We collected datasets consisting of weekly parasitologically confirmed malaria cases over an average of 10 years from health facilities in 10 districts of Ethiopia (Figure A1). The data arise from passive surveillance systems in selected districts for the years 1990–2000. Original data collected on the basis of Ethiopian weeks (which range from 5 to 9 days) were normalized to obtain mean daily cases for each Ethiopian week, and normalized data were used for all analysis. Data are summarized in Table 1.

**Table 1:** Characteristics of the study areas

District	Follow-up (y)	Daily microscopically confirmed cases			
		Mean	SD	Minimum	Maximum
Alaba	11.3	39.0	27.3	0	163.0
Awasa	7.7	11.3	11.0	0	77.4
Bahirdar	7.3	22.1	15.2	0	83.3
Debrezeit	11.2	25.3	25.8	0.9	146.7
Diredawa	9.8	25.3	29.5	0.4	329.9
Hosana	11.3	19.4	17.4	0.1	95.7
Jimma	10.3	13.2	14.0	0.3	85.3
Nazareth	9.3	17.7	16.0	0	109.3
Wolayita	9.3	13.9	12.1	0	113.1
Zeway	8.3	22.0	17.5	1.1	102.0

### Epidemic Detection Algorithms To Be Tested

We investigated four classes of algorithms for triggering alert thresholds. In each case, an alert was triggered if the defined threshold was exceeded for 2 consecutive weeks. (This choice is intended to improve the specificity of the alert system for any given threshold.) If another alert was triggered within 6 months, it was ignored, on the assumption that intervening after the first alert would prevent another epidemic within the next 6 months. For the purposes of historically based thresholds (1 and 2 below), the thresholds for each year were calculated on the basis of all other years in the dataset for a given health facility, excluding the year under consideration.

### Weekly Percentile

The threshold was defined as a given percentile of the case numbers obtained in the same week of all years other than the one under consideration. The use of percentile as alert threshold is straightforward, and the method is relatively insensitive to extreme observations.

### Weekly Mean with Standard Deviation (SD)

We defined the threshold as the weekly mean plus a defined number of SDs. Mean and SD were calculated from case counts, smoothed case counts, or log-transformed case counts.

### Slide Positivity Percentage

Some studies have indicated that the proportions of positive slides were significantly higher than the usual rate during epidemics (*16**,**17*), but whether the rise in proportion of positive slides occurs early enough to serve as a useful early detection system is not known. Slide positivity proportion was calculated from the number of blood slides tested and positive slides for malaria parasites.

### Slope of Weekly Cases on Log Scale

We hypothesized that rapid multiplication of the number of normalized cases from week to week might signal onset of an epidemic. To test this hypothesis and the usefulness of detecting such changes as a predictor of epidemics, we defined a set of alert thresholds on the basis of the slope of the natural logarithm of the number of normalized cases. An advantage of the slide positivity and log slope methods over the others is that they can, in principle, be used to construct alert thresholds in the absence of retrospective data.

### Comparison of Alert Thresholds

**Figure 1:**
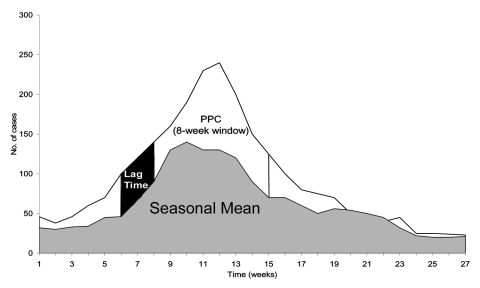
Method for calculating potentially preventable cases (PPC) by using weekly mean. PPC is obtained from cases in excess of the weekly mean with an 8-week window.

To circumvent the difficulties inherent in defining a "true" epidemic and to compare the properties of these thresholds on a scale that reflects the potential, operational uses of alert thresholds, we evaluated each alert threshold algorithm for the number of alerts triggered and the number of cases that could be anticipated and prevented ("potentially prevented cases") if that alert threshold were in place. Potentially prevented cases (PPC) for each alert were defined as a function of the number of cases in a defined window starting 2 weeks after each alert (to allow for time to implement control measures). The window of effectiveness was assumed to last either 8 or 24 weeks (to account for control measures whose effects are of different durations). Since no control measure would be expected to abrogate malaria cases completely, we considered two possibilities for the number of cases in each week of the window that could be prevented: 1) cases in excess of the seasonal mean and 2) cases in excess of the seasonal mean minus 1 SD. When the observed number of cases in a week is less than the seasonal mean or the seasonal mean minus the SD, PPC is set to a minimum value of zero for that week. Figure 1 depicts graphically how the PPC was calculated. For each value of each type of threshold at each health facility, the number of PPC was transformed into a proportion (percentage), by adding the number of PPC for the alerts obtained and dividing this sum by the sum of the number of potentially prevented cases, over all weeks in the dataset.

To compare the performance of dissimilar alert types on a single scale, a curve was plotted for each type of algorithm that showed mean percent of PPC (%PPC) over all districts versus average number of alerts triggered per year, with each point representing a particular threshold value. "Better" threshold types and values are those that potentially prevent higher numbers of malaria cases with smaller numbers of alerts.

### Random, Annual, and Optimally Timed Alerts

To evaluate the improvement in timing of alerts provided by each of these algorithms, we calculated PPC for alerts chosen on random weeks during the sampling period. We also made comparisons to two alert-generating policies that could not have been implemented but are in some sense optimal in hindsight. First, we evaluated a policy of triggering one alert each year on the "optimal" week, i.e., the week with the maximum value of PPC. The value of PPC corresponding to the optimal week simulated an "optimally timed" policy of annual interventions; thus, it represents one alert every year. Second, we retrospectively went through data for each site to identify the optimal timing of alerts if one had perfect predictive ability; namely, we compared PPC for a single alert generated on every week of the dataset and chose the optimal week for one alert; then we went through the remaining weeks and chose the optimal week for a second alert, and so on. This system allowed us to plot an upper bound curve for the best choice of alert times, given a defined alert frequency.

## Results

The dataset consists of a total of 687,903 microscopically collected malaria cases from a health facility in each of 10 districts over an average of 10 years. On average, each of the 10 health facilities treated 11–39 malaria cases daily and >300 cases per day during the peak transmission season (Table 1). In most districts, including Awasa, Zeway, Nazareth, Jimma, Diredawa, Debrezeit, and Wolayita, the number of cases showed clear seasonal fluctuation over time. Alaba, Bahirdar, and Hosana showed longer term variation, with an increasing trend in Alaba and more complex patterns in Hosana and Bahirdar. The number of cases in all districts shows a clear year-to-year variation.

**Figure 2:**
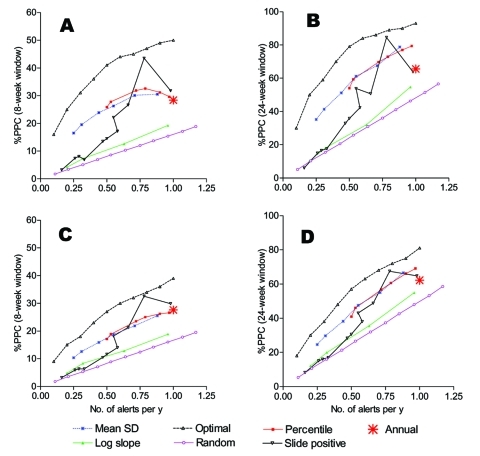
Percent of potentially preventable cases (PPC) by number of alerts per year for different algorithms. (A) and (B) were obtained from cases in excess of the weekly mean with window of effectiveness of 8 and 24 weeks, respectively. (C) and (D) were obtained from cases in excess of the weekly mean minus one SD for window of 8 and 24 weeks, respectively. The scale of y-axis is higher for (B) and (D) because they are based on 24 weeks of PPC (based on the random alert, the %PPC for the 24-week window is three times that of the 8-week window of effectiveness).

**Figure A2:**
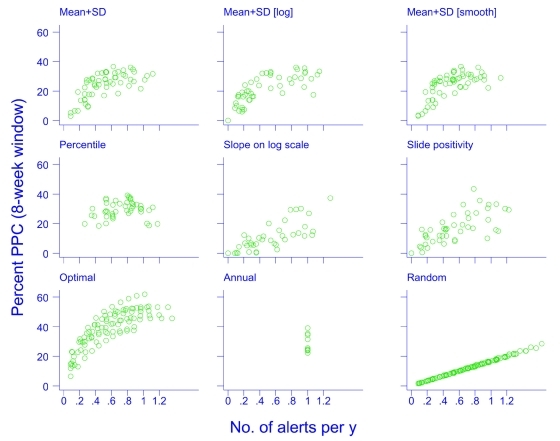
Percent potentially preventable cases (PPC) by number of alerts per year from all districts for each alert threshold algorithm.

The number of alerts triggered and %PPC obtained for each level of a threshold by type of algorithm varied in the 10 districts (Figure A2). Number of alerts triggered and %PPC for a single alert threshold level are represented by a point. These points are summarized in Figure 2, which compares the performance of all algorithms on a single scale and explores the sensitivity of results to the choice of function for determining PPC [reducing cases to weekly mean, (A) and (B), or weekly mean minus 1 SD, (C) and (D)] and the choice of window of effectiveness [8 weeks, (A) and (C); 24 weeks, (B) and (D)]. All alert threshold algorithms potentially prevented a larger number of cases than random alerts, whose performance is shown as a straight line with cases increasing in proportion to the number of alerts.

The alert threshold algorithm based on percentile performed as well as or better than the other algorithms over the range of number of alerts triggered that we examined. For a given number of alerts triggered, it prevented a greater %PPC compared to other methods. Relative to optimally timed alerts, the percentile algorithm performed well, within 10% to 20% of the best achievable performance. The slope on log scale algorithm performed slightly better than the random but much worse than the other algorithms.

Threshold algorithms defined as the weekly mean plus SDs based on different forms of the data (normalized case counts, smoothed case counts, or log-transformed case counts) performed similarly, except that the algorithms based on the smoothed cases and log-transformed cases triggered fewer alerts at a given threshold value compared to the algorithm based on normalized cases.

For highly specific threshold values (triggering relatively few alerts), the slide positivity proportion showed a lower %PPC than any other algorithm except the log slope. This pattern was reversed at more sensitive threshold values; slide positivity thresholds of <65% showed a higher %PPC than the other threshold methods for a given number of alerts per year.

The annual alert, which corresponds to intervening every year during a fixed optimal week (generally just before the high transmission season), prevented 28.4% of PPC. However, an equivalent %PPC was prevented by the weekly mean and percentile algorithms with only 0.5 alerts per year.

The preceding numbers refer to the weekly mean with 8-week window assessment (Figure 2A). Comparative performance of the different alert thresholds was insensitive to the length of the window and the choice of function to define potentially prevented cases (Figures 2A–D). In all cases, the percentile algorithm performed best overall, although the difference became smaller for the 24-week window.

**Figure 3:**
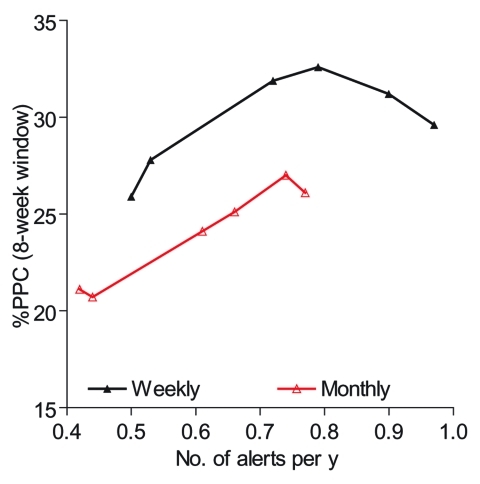
Percent of potentially preventable cases (PPC) obtained using weekly and monthly data with an 8-week window.

In all alert threshold algorithms, the %PPC rises with increasing number of alerts and then levels off approximately at 0.4 to 0.6 alerts per year. The interrelationship between levels of percentile used, number of alerts triggered, and %PPC is presented in detail to illustrate the factors that would contribute to choosing a cost-effective threshold value. Table 2 shows that 85th percentile as a threshold level triggered 0.72 alerts per year with 31.9% of PPC; 80th and 75th percentiles, on the other hand, gave 0.79 and 0.9 alerts per year with 32.6% and 31.2% of PPC, respectively. For an additional 0.1–0.2 alerts per year, the gain is nil. Similarly, 70th percentile with approximately one alert every year resulted in even fewer potentially prevented cases (29.7% of PPC). Most of the possible maximum PPC can be achieved by using a weekly percentile alert threshold that can only trigger 0.4–0.6 alerts per year, and threshold based on 85th to 90th percentile trigger, on average, similar alerts per year. Figure 3 shows that alert threshold methods based on weekly data perform much better than those based on monthly data.

**Table 2:** Potentially preventable cases (PPC) by level of the seasonal percentile threshold in relation to number of alerts per year (8-week window)

District	Six alert threshold levels based on seasonal percentile											
	95th percentile		90th percentile		85th percentile		80th percentile		75th percentile		70th percentile	
	No. alerts	% PPC	No. alerts	% PPC	No. alerts	% PPC	No. alerts	% PPC	No. alerts	% PPC	No. alerts	% PPC
Alaba	0.44	18.6	0.53	20.1	0.62	24.5	0.62	23.4	0.8	28.8	0.97	30.1
Awasa	0.55	28.1	0.65	28.1	0.65	35.6	0.91	32.9	0.91	32.9	1.0	20.2
Bahirdar	0.55	27.9	0.55	27.9	0.55	27.9	0.82	37.6	0.82	38.4	0.82	38.4
Debrezeit	0.27	19.8	0.54	28.5	0.54	37	0.54	36.2	0.54	36.2	0.8	39
Diredawa	0.61	25.2	0.61	26.6	0.82	26.9	0.82	26.9	1.1	31	1.1	31.1
Hosana	0.35	25.6	0.62	32.6	0.71	33.5	0.8	34.3	0.97	28	0.97	28
Jimma	0.39	24.9	0.39	24.9	0.78	31.8	0.87	33.1	0.97	32.1	0.87	31.9
Nazareth	0.54	33.9	0.54	33.9	0.86	34	0.86	34	1.1	18.7	1.2	19.7
Wolayita	0.54	24.8	0.54	24.8	0.86	30.5	0.86	30.5	0.97	29.9	1.1	29.9
Zeway	0.36	30.2	0.36	30.2	0.84	37.2	0.84	37.2	0.84	36.4	0.84	28.5
Total	0.46	25.9	0.53	27.8	0.72	31.9	0.79	32.6	0.9	31.2	0.97	29.7

## Discussion

We have described a novel method for evaluating the performance of malaria early detection systems for their ability to trigger alerts of unusually high malaria case numbers with sufficient notice so that control measures can be implemented in time to have an effect on the epidemic. By defining the performance of an algorithm in terms of the potentially prevented cases falling in a given time window after the alerts are generated, we attempted to capture the public health value of an alert system, which is its ability to predict excess malaria cases. Given the same number of alerts triggered by different potential detection algorithms, the objective is to identify an alert threshold algorithm that triggers alerts at the beginning of unusually high transmission periods, on the assumption that such periods are the ones in which interventions are likely to prevent the most cases.

Given the wide variations in malaria transmission, no standard expectation exists about what proportion of cases can be averted with what intervention. With the assumption that the magnitude of the effect of an intervention would be related to the difference between the observed number of cases and size of the long-term seasonal mean and SD, we calculated PPC. In other words, we assumed that an intervention would lower the number of cases towards the underlying seasonal mean or, if very effective, to l SD below the underlying mean. The sensitivity of the relative performance of the different algorithms was tested by using different window periods (8 or 24 weeks) of effectiveness of possible intervention methods. These window periods are based on the duration of effects of common interventions, such as insecticide spraying, which have residual activity of 8 to 24 weeks (*18**–**20*), and other emergency malaria epidemic control measures such as mass drug administration that could lower the incidence of malaria within an 8- to 24-week range (*21*). Unlike the complex detection algorithms tested for other diseases (*22**–**26*), the algorithms compared in this study are simple to implement without the use of computers, which are currently unavailable to malaria control efforts in most parts of Africa.

At relatively smaller number of alerts triggered, threshold algorithms based on percentile anticipated the highest percentage of the potentially preventable malaria cases of all approaches. The percentile algorithm's good performance relative to the optimally timed alerts indicates that it triggers alerts at the beginning of epidemics rather than in the middle of ongoing epidemics. Given the attractive characteristics of the percentile algorithm, a further question is what percentile level one should use. Beyond 0.4 to 0.6 alerts/year, the %PPC leveled off because most of the peaks with higher numbers of cases, possibly epidemic periods, were detected with fewer alerts by using 85th to 90th percentiles. The leveling off of %PPC occurs because we assume that an alert triggered at week t, which leads to application of intervention measures, will prevent another alert until week t + 24. In practical terms, an intervention initiated after an alert was triggered by a less-specific alert threshold during relatively lower transmission might provide little benefit for a community in reducing malaria transmission, especially if it consumed scarce resources that would then be unavailable during periods of higher transmission.

In situations in which cost is not an issue and yearly application of preventive measures is possible, slide positivity proportion could be recommended. It performed as well as or better than all types of algorithms when all algorithms were set to trigger an average of one alert per year. During malaria epidemics, the slide positivity proportion becomes very high (*16**,**17*), and the rise in the proportion of positive slides may begin at the onset of the epidemic to give an early warning, as our data showed. The interannual variation in the time and intensity of the peak of malaria transmission impacts the effectiveness of the annual alert with interventions at a fixed week every year; using the slide positivity proportion would identify the right time for intervention. The limitation for using slide positivity proportion is that it requires evaluating the cut-off level in individual health facilities and revising the baseline with a change of health personnel because the baseline slide positivity proportion may vary due to differences in epidemiologic patterns of malaria and other causes of fever. Thus, although slide positivity proportion thresholds could be defined in the absence of historical data, our results suggest that such data would be required to calibrate the threshold properly for any given locality. The slope on log scale algorithm performed poorly because the largest proportional rate-of-change for the number of cases tended to occur during periods of very low case numbers (perhaps reflecting chance fluctuations).

Comparative performance of different alert thresholds was insensitive to the length of the window and the choice of function to define potentially prevented cases. This study indicated the use of weekly data rather than monthly data in constructing threshold methods and in follow-up prevented more cases, consistent with the World Health Organization's recommendations (*5*).

A key limitation of our study was that the use of a long-term measure of disease frequency from a retrospective dataset assumes that the long-term trend did not change significantly and that the method of data collection remained the same. Factors such as change of laboratory technician affect the number of slides that are judged positive for malaria parasites. Such changes should be considered, and revising the threshold values frequently with the most recent data and standardized training of laboratory technicians are advisable. Moreover, existing interventions (which may, in some places, have been based in part on algorithms of the sort we considered) could also interfere with the trend. In this analysis, we did not exclude epidemic years from the data since, on the one hand, we do not have a standard definition of malaria epidemics and, on the other hand, all possible data points should be used to calculate measures of disease frequency and scatter to come up with potential threshold levels unless the data points were considered as outliers.

We deliberately chose to evaluate only simple, early detection algorithms, rather than more complex ones that might require climate or weather data or complicated statistical models. In the dataset we considered, the best of these simple algorithms performed quite well relative to the best possible algorithm, which suggests that they may be adequate for many purposes. In principle, the method we propose could easily be applied to evaluate more complex, early warning algorithms and to test whether their added complexity results in substantially better performance. It is an open question whether the same methods would work as well in localities (or for diseases) with different patterns of variation in incidence, for example, in those with less pronounced seasonal peaks in incidence.

In conclusion, we have shown that simple weekly percentile cutoffs appear to perform well for detecting malaria epidemics in Ethiopia. The ability to identify periods with a higher number of malaria cases by using an early detection method will enable the more rational application of malaria control methods. The comparative technique developed in this study may be useful for testing other proposed alert threshold methods and for application in other populations and other diseases.

## Appendix

### Data

A team from the Ethiopian National Malaria Control Program visited different health facilities to look for complete epidemiologic data with consistent malaria case definitions. After the field trip, health facilities with relatively high-quality recording and information systems were chosen. Health personnel from each facility selected for the study and staff from the National Malaria Control Program were given training on compiling data on illness and death from the existing patient logs.

Raw data for each week (*j*) of each year (*i*) from each health facility (*h*) consist of the number of slides examined during that week (*E_hij_*) and the number of those slides that were positive for *Plasmodium falciparum* (*C_hij_*). The original data were collected weekly on the basis of the Ethiopian calendar, which consists of 12 months, each with three 7-day weeks and one 9-day week (48 weeks), and a 49th week of 5 days. Weekly numbers of cases were normalized to a daily average by dividing raw case numbers by the number of days in the week.

### Notation

Each point in the dataset refers to some measure of malaria prevalence in a given health facility (*h*) during a given week. Data from a given health facility during a given week may be indexed by an absolute time (*t*), or by a year (*i*) and a week of the year (*j*=1…49), to facilitate comparisons of the same week across years.

### Subscripts

*h* – health facility*t* – time in weeks*i* – year*j* – week of the year, using the Ethiopian calendar*f_t_* = number of days in week *t*

### Weekly Data and Their Transformations

*E_ht_* – number of slides examined at health facility *h* in week *tC_ht_* – number of positive slides ("cases") at health facility *h* in week *tX_ht_* = *E_ht_* / *f_t_* – normalized daily average total slides examined for week *t* at health facility *hY_ht_* = *C_ht_* / *f_t_* – normalized daily average positive slides for week *t* at health facility *hL_ht_* = ln(*Y_ht_*) – log-transformed cases for week *t* at health facility *hS_ht_* =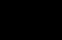
– order-3 moving average daily cases for week *t* at health facility *hP_ht_* = 100%* *Y_ht_∕X_ht_* – slide-positivity percentage for week *t* at health facility *h*Z*_hj_* – number of data points (years) for week *j* in dataset from health facility *hn_h_ –* total number of weeks in dataset from health facility *h*

### Threshold Definitions

T*_hijs_* – threshold value in health facility *h* during week *j* of year *i*, using sensitivity *s*Φ*_hTs_* – number of alerts generated in health facility *h* by threshold type *T* with sensitivity *sQ_phij_ –* the *p*th percentile of *Y_h.j_* excluding year *is* – sensitivity of a threshold: number of standard deviations (SD), percentile cutoff, threshold for slide positivity, or log slopeμ*_hij_* =[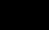]∕ [ Z*_hj_* – 1], weekly mean of normalized cases for week *j*, from all years except *i*σ_Y_*_hij_* =√ {[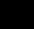
(Y*_hij_* – μ*_hij_*)^₂^]∕[ Z*_hj_* – 2]}, weekly SD of normalized cases for week *j*, from all years except *i*

## Mathematical Details Used To Calculate Epidemic Detection Algorithms

### Weekly Percentile

Threshold is exceeded when *Y_hij_* > *T_hij_* , where *T_hij_* =*Q_phij_*, where *Q_phij_* represents the *p*th percentile (*p* = 70, 75, 80, 85, 90, or 95) percentile of observations from week *j* at facility *h* in years other than *i*.

### Weekly Mean with SD

Threshold is exceeded when *Y_hij_* > T*_hij_*, where T*_h_*_ji_ = µ*_hij_* + βs_Y_*_hij_*, where β = 0.5, 1.0, 1.5, 2.0, 2.5, or 3. A parallel definition is used for log-transformed (*L_hij_*) and smoothed (S*_hij_*) data, by using corresponding means and SD.

Normalized counts: the number of normalized weekly cases was used to derive the weekly mean and SD.

Smoothed normalized counts: To improve data smoothness, moving averages *S_hij_* were obtained (see Notation above) from normalized counts and used both to calculate mean and SD for the thresholds, and to compare against the thresholds. Weekly means and SD were calculated from the {*S_hij_*}.

Log-transformed series: To obtain data with reduced right skew, logged weekly counts *L_hij_* were obtained (see Notation above) from normalized counts and used both to calculate mean and SD for thresholds and to compare against the thresholds. Weekly means and SD were calculated from the {*L_hij_*}.

### Slide Positivity Percentage

Slide positivity proportion (*P_ht_*) was calculated for each week:*P_ht_* = 100% * *Y_ht_*∕*X_ht_*Threshold is exceeded when *P_ht_* > *z*, where *z* = 30%, 35%, 40% …80%.

### Slope of Weekly Cases on Log Scale

We defined a set of alert thresholds based on the slope (*M_ht_*) of the natural logarithm of the number of normalized cases: *M_ht_ = L_ht_ – L_ht-1_*

The threshold is exceeded when *M_ht_* >*m*, where *m* = 0.2, 0.3, 0.4, or 0.7, which approximately corresponds to 25%, 35%, 50% or 100% increase relative to previous week’s number of cases.

### Generating Potentially Prevented Cases

Potentially prevented cases (PPC) for each alert were defined as a function (*q*) of the number of cases in a defined window starting 2 weeks (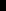) after the alert (to allow for time to implement control measures). The window of effectiveness (∞) was assumed to last either 8 or 24 weeks (to account for control measures whose effects are of different durations). Since no control measure would be expected to abrogate malaria cases completely, we considered two possibilities for the number of cases in each week of the window that could be prevented: 1) cases in excess of the weekly mean: *q*_1_*_ij_* = max(0,*Y_ij_* - *µ_ij_*), and 2) cases in excess of the weekly mean minus l SD: *q*_2_*_ij_* = max(0,*Y_ij_* – [*µ_ij_ - σ_Yij_*]), where *Y_ij_* is the observed number of cases, and *µ_ij_* and *σ_Yij_* are mean and SD for week *j* excluding year *i*. When the observed number of cases in a week is less than the weekly mean (in calculating *q*_1_) or the weekly mean minus the SD (in calculating *q*_2_), *q* is set to a minimum value of zero for that week. The PPC using a threshold type *T* with sensitivity *s* in dataset from health facility *h*, using function *q_k_* is written as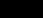:1) *PPC*^1^*_h_*_Ts_ = 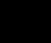
max (0, Y*_h_*_ji_ - m_hji_)2) *PPC*^2^*_h_*_Ts_ = 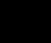
max (0, Y_hji_ – [m_hji_ - s_Y_*_h_*_ji_]),

where Φ_hTs_ = number of alerts triggered by threshold type *T* with sensitivity s in data set from health facility *h*; *t*
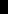
= the time of alert φ; 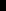 = 2 representing number of weeks after alert turns on; and ∞ = 8 or 24 representing window period. Once an alert threshold triggers an alert at time *t*, and a control measure is applied, we ignored alerts within the next 6-month period until *t*+24 with the assumption that effective control measures will be taken and risk for another epidemic soon will be minimal.

For each value of each type of threshold at each health facility, the number of potentially prevented cases was transformed into a proportion (percentage), by adding the number of potentially prevented cases for the alerts obtained and dividing this sum by the sum, over all weeks in the dataset, of the number of potentially prevented cases in that week. Let %PPC*_h_*_Ts_ denote percent of PPC*_h_*_Ts_ and %PPC_Ts_ denotes the mean of %PPC*_h_*_Ts_ from the different health facilities.1) %*PPC*^1^*_h_*_Ts_ =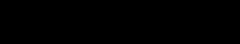
∕
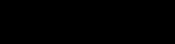
2) %*PPC*^2^*_h_*_Ts_=



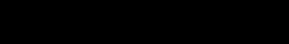



∕
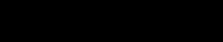

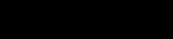


(Note: here the t and ij notations are used interchangeably.)

To compare the performance of dissimilar alert types on a single scale, a curve was plotted for each type of algorithm showing mean %PPC vs. average number of alerts triggered per year, with each point representing a particular threshold value.

### Random Alert

To calculate the expected PPC for randomly timed alerts, the excess cases under excess case definition (*q_k_*) for all weeks in the study was averaged to obtain an overall mean. For a window of length ∞ weeks, PPC*_h_*_rΦ_ represents the expected PPC for Φ randomly chosen alerts in dataset from health facility h.




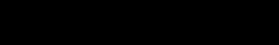







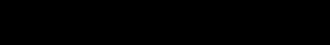




### Annual Alert

To determine the optimal week, we calculated PPC for a policy of triggering an alert automatically during week *j* (*j* = 1..49) every year, using window ∞. The optimal week was the week *j* with the maximum value of PPC. The value of PPC corresponding to the optimal week simulated an "optimally timed" policy of annual interventions; thus, it represents one alert every year.


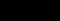
 describes PPC from health facility *h* when the alert is triggered every year at week *j*, and 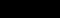 denotes the maximum PPC from the best possible week.


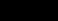
 = 
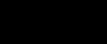
and 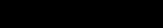



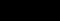
 = 
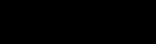
 and 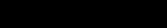


### Optimally Timed Alerts

To calculate the expected PPC for optimally timed alerts, we followed a recursive procedure. First, we searched through all weeks in the data set and chose the single week on which an alert would have the maximum PPC under a given case definition (*q_k_*). Then, the process was repeated with the weeks that remained after "blocking" alerts for a period of 24 weeks before or after the first alert (since our algorithms were similarly constrained never to have two alerts <24 weeks apart). This process was repeated up to a total of 10 alerts for each site. This process approximates the optimal timing of alerts, although theoretically all possible combinations of a given number of alerts would have to be tried to ensure optimal timing. Since each site had a slightly different number of weeks in the dataset, a given number of alerts corresponded to slightly different frequencies in the different sites (hence, the horizontal scatter of points in Figure 2). The "optimal alert" points in Figure 2 were calculated by "binning" similar frequency values and averaging the %PPC across values in a bin. Percent PPC from all districts for a given number of random and optimally timed alerts and for the annual alert were calculated in analogous ways.

### Comparison of Use of Weekly versus Monthly Data

We also compared the efficiency of the weekly percentile method applied to weekly data vs. the same method applied to monthly data. For this purpose weekly data were converted into monthly data, and alert threshold levels based on the percentile were built and a similar procedure was used, except that an alert was triggered when the observed monthly value exceeded the threshold determined by the method in any single month. For this comparison, we considered PPC formula *q*_1_, with φ = 8 weeks. A set of computer programs written in Stata to perform the methods presented in this article is available.
